# Comparison of clinical outcomes with InterTan vs Gamma nail or PFNA in the treatment of intertrochanteric fractures: A meta-analysis

**DOI:** 10.1038/s41598-017-16315-3

**Published:** 2017-11-21

**Authors:** Jian-xiong Ma, Ming-jie Kuang, Zheng-rui Fan, Fei Xing, Yun-long Zhao, Lu-kai Zhang, Heng-ting Chen, Chao Han, Xin-long Ma

**Affiliations:** 1 Biomechanics Labs of Orthopaedics Institute, Tianjin Hospital, Tianjin, 300050 People’s Republic of China; 2 Department of Orthopedics, Tianjin Medical University General Hospital, Tianjin, 300052 People’s Republic of China

## Abstract

Intertrochanteric fractures are common injuries in the elderly. Conventional intramedullary nails including Gamma 3 locking nail and proximal femoral nail antirotation (PFNA) were designed for unstable intertrochanteric fractures. The InterTan (IT) nail system, introduced in 2005, has been reported superior biomechanical and clinical outcomes compared with 1-screw nailing system. However, some recent studies have reported that IT did not improve functional recovery in patients with intertrochanteric fractures. Randomized controlled trials (RCTs) or prospective cohort studies were included in our meta-analysis. We used the PRISMA guidelines and Cochrane Handbook to evaluate the quality of included studies to ensure that the pooled data of our meta-analysis were reliable and veritable. Our pooled data analysis demonstrated that IT was as effective as the control group in terms of Harris Hip Score (HHS), blood loss, total complications, union time, length of hospital stay, revision rate, and fluoroscopy time. IT shows less implant cut-out rate and femoral fractures when compared with control groups.

## Introduction

Intertrochanteric fractures are common injuries and often occurred in elderly patients. Several studies reported that an annual incidence of intertrochanteric fractures are more than 150,000 in the United States^1,2^. Over the past decades, the dynamic hip screw (DHS) as an extra-medullary stabilization device has been the most widely used implant. However, complications such as hip varus deformity, delayed union and loosen screws have been frequently reported with DHS fixation particularly in unstable fractures^3,4^. Conventional intramedullary nails including gamma 3 locking nail and proximal femoral nail antirotation (PFNA) were designed for unstable intertrochanteric fractures^5^. Recently, the intramedullary nails showed better functional recovery and a better biomechanical stability compared with DHS^6,7^.

The InterTan Nail (IT), using an integrated 2-screw system, provide increased stability and resistance to femoral head rotation and decrease cut-out rate when compared with the conventional intramedullary nails such as PFNA and Gamma nails. A study compared the biomechanical stability between the IT and Gamma 3 showed that IT group provided better anti-compression and anti-rotation stability in an intertrochanteric fractures biomechanical model^8^. A prospective clinical study reported that IT showed better clinical outcomes including varus angle, union time, full weight bearing, and cut-out rate than PFNA with 1-year follow-up^9^. A randomized controlled trial (RCT) with five-years of follow-up demonstrated that the IT only performed better in functional outcome and hospital stay the 6-month follow-up, however, 33/104 patients were available for the final 5 year follow-up and no significant differences were recorded between two groups^10^. Therefore, it is necessary to investigate whether IT leads to better postoperative functional recovery for intertrochanteric fractures compared with the conventional intramedullary nails.

## Materials and Methods

### Search strategy

**Figure 1:**
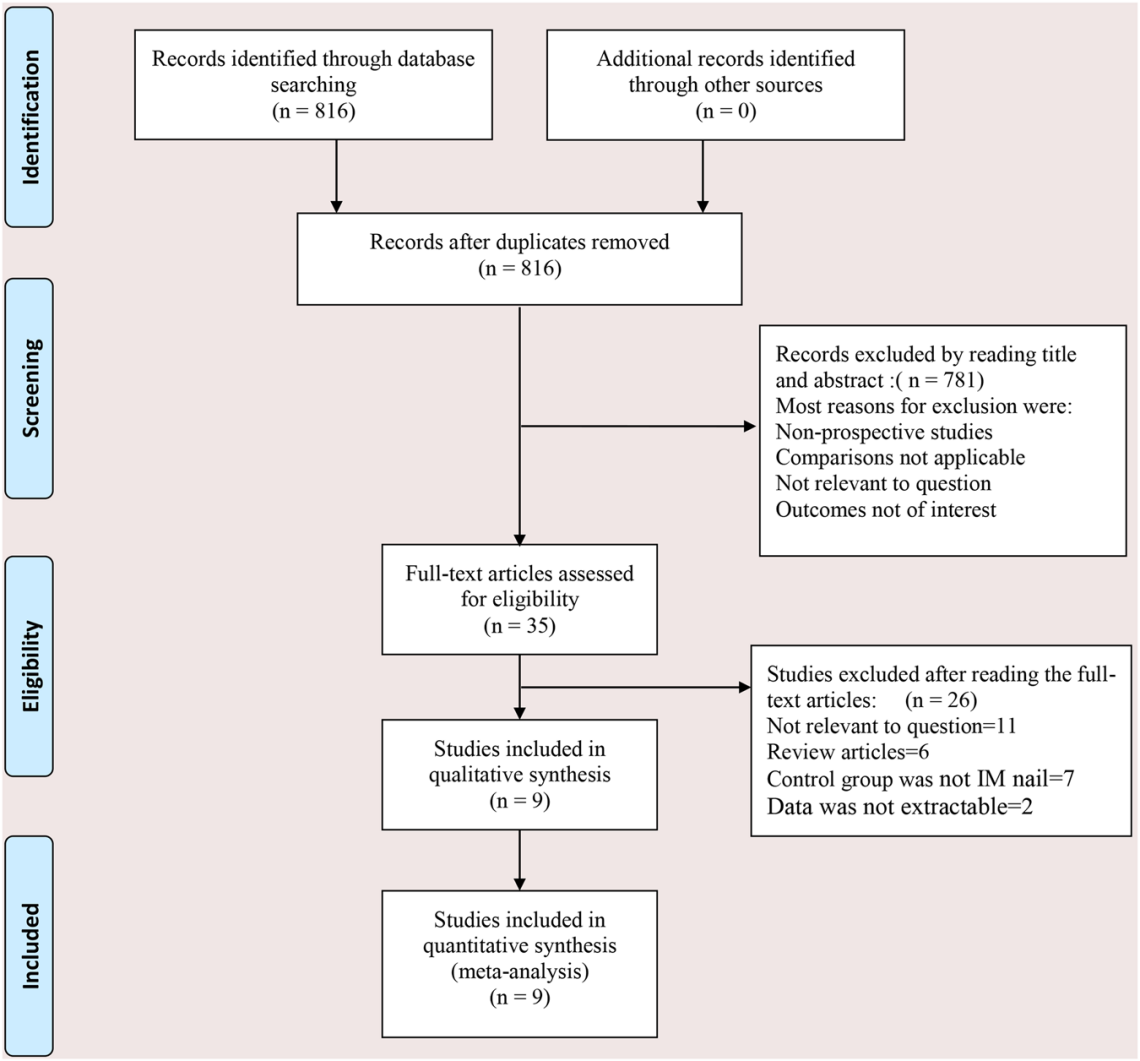
Search results and selection procedure.

We used the PRISMA guidelines^11^, GRADE system^12^ and Cochrane Handbook^13^ to evaluate the quality of the included studies to make sure the data reliable and veritable. A systematic review and meta-analysis was conducted to identify all studies from data base involving IT and conventional intramedullary nails for intertrochanteric fractures in electronic databases including Web of Science, Embase, PubMed, the Cochrane Controlled Trials Register, and the Cochrane Library up to May 2017. Only RCTs and prospective cohort studies performed on human beings is included. The search strategy was presented in Supplemental Table 1. Flow chart of the trial selection process was presented in Fig. 1. In addition, we also conducted other databases according to the Cochrane Collaboration Guidelines.

### Inclusion and exclusion criteria

Included studies were considered eligible if they met the PICOS criteria as follows:

Population: Patients with intertrochanteric fractures

Intervention: 2-screw intramedullary nail (IT)

Comparator: 1-screw intramedullary nails (PFNA or Gamma nail)

Outcomes: The primary outcomes included: Harris Hip Score (HHS). Secondary outcomes contained: union time, blood loss, postoperative complications, length of hospital stay, implant cut-out, femoral fractures, revision surgery, varus angulation, fluoroscopy use, and surgery time.

Study design: RCTs or prospective cohort studies or retrospective cohort studies with prospective collected data.

Only published clinical studies were included; the included studies were required to contain at least one main patient reported outcome. Two professional authors screened the relevant literature independently. A consensus was reached through discussion, once disagreement was existed between authors.

Exclusion criteria were review articles, studies with insufficient outcome data and studies not prospective collected data.

### Data extraction

A standard data extraction form was used to collect the relevant data from included studies. The relevant data included study location, main authors, sample size, study design, publishing date, gender, population, type of nails, age, follow-up, interventions, and patient reported outcomes. To make sure the extracted data was integrated, we contacted the corresponding authors of the included studies and to get any missing data. Two reviewers extracted the data independently. If there were disagreements between two authors, consensus was reached through discussion.

### Risk of bias and quality assessment

**Figure 2:**
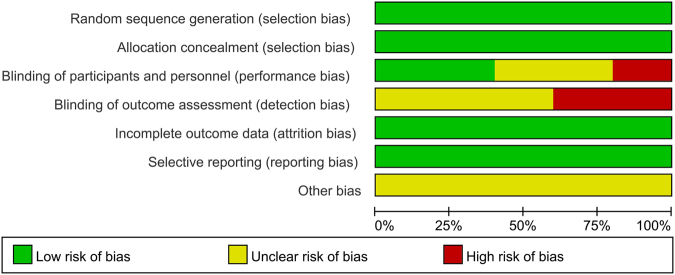
Risk of bias graph: review authors’ judgements about each risk of bias item presented as percentages across all included studies.

**Figure 3:**
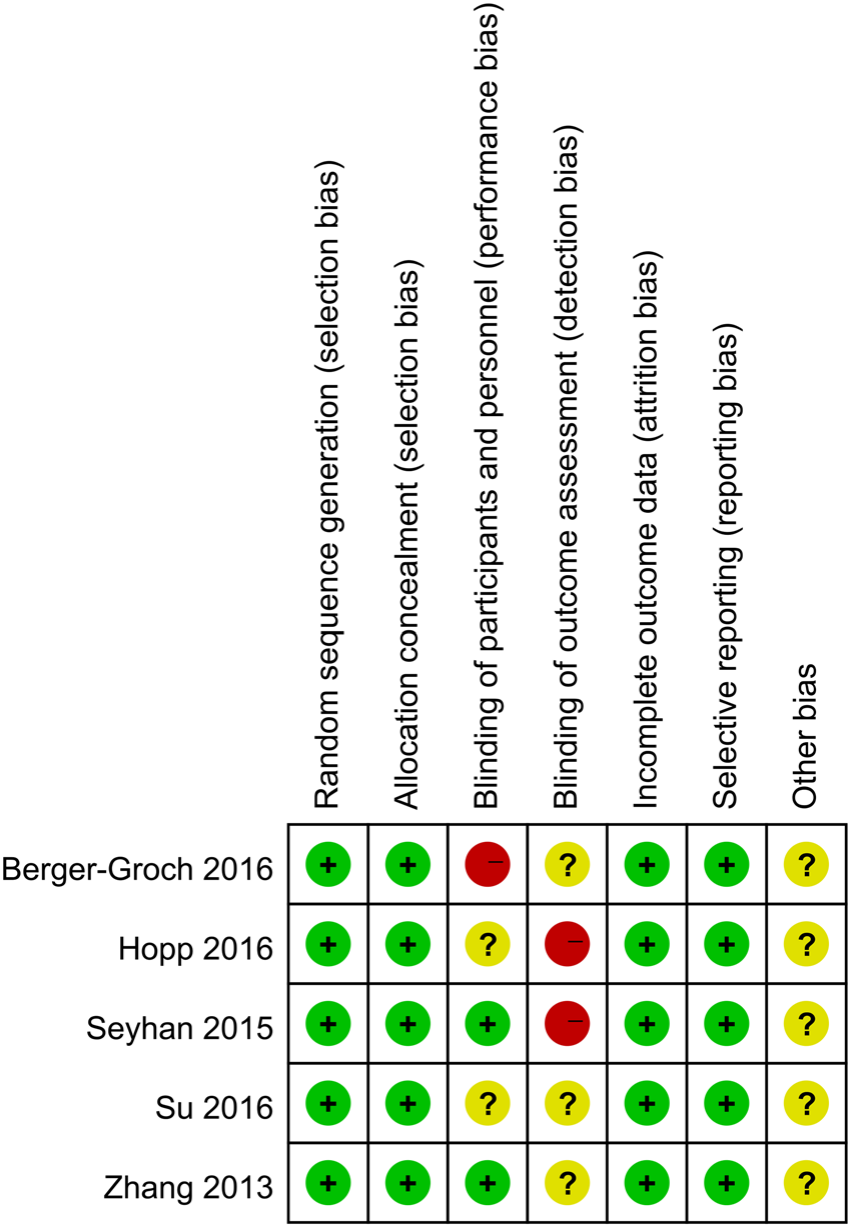
Risk of bias summary: review authors’ judgements about each risk of bias item for each included study.

According to the Cochrane Handbook for Systematic Reviews of Interventions, the methodological quality and basis of the included RCTs were assessed as follows: randomization, allocation concealment, blind method, selective reporting, incomplete outcome data, and other bias (Figs 2 and 3). For cohort studies, we used an eight-point Newcastle-Ottawa Scale (NOS)^14^ to assess the quality of cohort studies. When the quality score was greater than five points, the included studies were considered to be of high quality^15^. The risk of bias was evaluated by eight items. (Supplemental Table 2 presented the eight items in detail**)**.

### Assessment of reporting bias

We used funnel plot to evaluated reporting biases qualitatively. Egger’s test was conducted in our meta-analysis to assess the reporting biases quantitatively.

### Data Extraction

All the data was presented as the form of mean ± standard deviation (SD) in our meta-analysis. All the methods were used to calculate the means ± SD on the basis of the *Cochrane Handbook for Systematic Reviews*.

### Statistical analysis and data synthesis

Meta-analyses was performed with Review Manager 5.3. We used the mean difference (MD) to evaluate the continuous outcomes such as HHS, union time, length of hospital stay, fluoroscopy time, blood loss, and surgery time with a 95% confidence interval [CI]. Relative risks (RR) with a 95% CI were used to assess dichotomous outcomes. We used the inverse variance and Mantel–Haenszel methods to combine separate statistics. If *P* values were less than 0.05, the results were considered statistically significant.

### GRADE the evidence

We used GRADE system to evaluate the level of the evidence and strength of recommendations for included outcomes. GRADE software was used to evaluate the evidence of included outcomes. Initially, RCTs were considered as high confidence in an estimate of effect and cohort studies were considered as low confidence in an estimate of effect. Reasons that may decrease level of confidence including rating limitations in study design or execution, rating inconsistency in results, indirectness of evidence, rating imprecision of results, and selective publication of studies. Reasons that may raise the level of confidence include large magnitude of the effect, rating the dose-response gradient, and rating the influence of all plausible residual confounding. The GRADE evidence were divided into four categories and the results of GRADE evidence was presented in Supplemental Table 3.

### Investigation of heterogeneity

Statistical heterogeneity of the included researchs were evaluated by chi-square test in accordance with *P* and I^2^. If the I^2^ < 50% and *P* > 0.1, the heterogeneity might not be important.We used fixed-effects model to evaluate relevant outcomes. If I^2^ was between 50% to 100%, it may represent substantial heterogeneity. A random-effects model was used to evaluate these outcomes. Meanwhile, we performed subgroup analysis and sensitivity analysis to interpret the potential source of heterogeneity.

## Results

### Search results

Initially, 816 citations were identified from electronic journals databases, of which 781 records were removed by primary screening. We read the full text of 35 remaining studies, 26 articles were excluded according to the inclusion and exclusion criteria. Finally, 9^9,10,16–22^ articles with 1119 patients that compared 2-screws with 1-screw intramedullary nail were included. All the included articles were published between 2013 and 2016. The data and characteristics of 9 included articles were summarised in Table 1.

**Table 1:** The characteristics of included studies.

Study(year)	Type of nails	2-screw group/1-screw group							Follow-up (mean)	Reference type
		Cases	Age	Gender	Fracture type (number)			ASA score		
			(mean)	(% male)	AO/OTA-A1	AO/OTA-A2	AO/OTA-A3			
Berger-Groch 2016	InterTan vs Gamma3	55/49	81.6/82	21.8/24.5	14/14	41/35		2.7/2.7	5 years	RCT
Hopp 2016	InterTan vs Gamma3	39/39	82.7/80.7	18/33.3	0/0	28/39	11/13	2.83/2.77	1 years	RCT
Seyhan 2015	InterTan vs PFNA	32/43	75.3/75.9	25/25.6	7/11	13/16	12/16	N/A	1 years	RCT
Su 2016	InterTan vs Gamma3	50/50	71.1/71.3	42/38	0/0	40/41	41/9	2.68/2.7	1 years	RCT
Wang 2013	InterTan vs PFNA	20/36	73.5/76.8	55/47.2	2/7	13/26	5/3	N/A	4.6 months	CS
Wu 2014	InterTan vs Gamma3	87/174	71.4/72.6	27/24.7	0/0	72/146	15/28	2.45/2.48	1 year	CS
Yu 2016	InterTan vs PFNA	75/75	75.2/74.2	44.4/46.6	0/0	40/35	35/37	N/A	1.7 years	CS
Zehir 2015	InterTan vs PFNA	102/96	76.8/77.2	38.2/38.5	0/0	93/92	9/4	N/A	1 years	CS
Zhang 2013	InterTan vs PFNA	57/56	72.9.72.4	34/40.3	0/0	45/45	12/11	2.47/2.57	1 years	RCT

AO/OTA: Arbeitsge-meinschaft für Osteosynthesefragen/ Orthopaedic Trauma Association, N/A: Not Applicable, ASA: American Society of Anesthesiologists, RCT: Randomized Controlled Trial, CS: Cohort Study, PFNA: Proximal Femoral Nail Antirotation.

### Results of meta-analysis

We performed subgroup analysis to explore the source of heterogeneity. The included studies were divided into two subgroups to minimize the heterogeneity.

### Primary outcome

#### HHS

**Figure 4:**
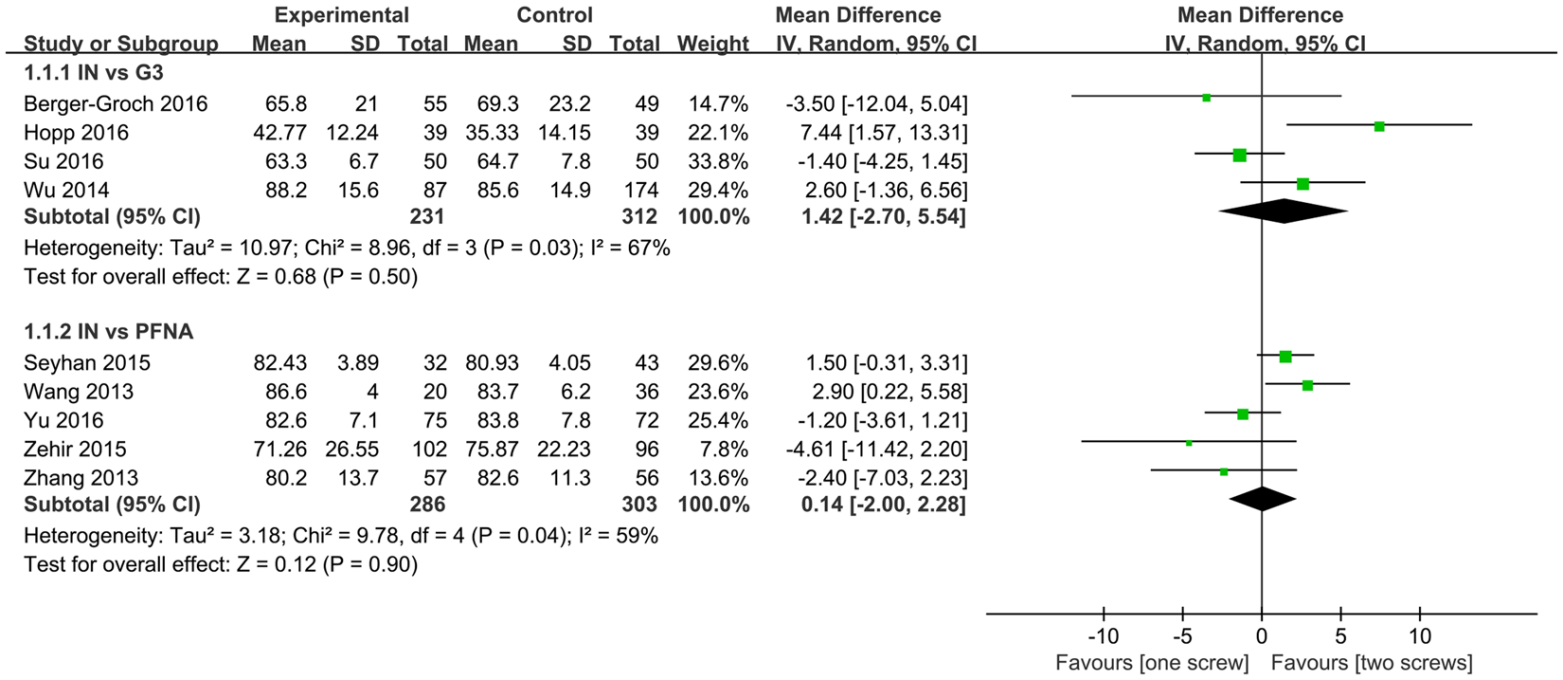
A forest plot diagram showed the HHS.

All the included nine studies assessing 1119 patients reported the HHS postoperatively. The single screw group was divided into two subgroups (PFNA and Gamma 3). No significant difference was found in IT vs. Gamma 3 (MD = 1.41, 95%CI:[−2.76, 5.59], P = 0.51; Fig. 4) and IN vs. PFNA (MD = 0.14, 95%CI:[−2, 2.28], *P* = 0.9; Fig. 4) subgroup. Random-effect model was used to explain the statistical heterogeneity.

### Secondary outcomes

#### Union time

**Figure 5:**
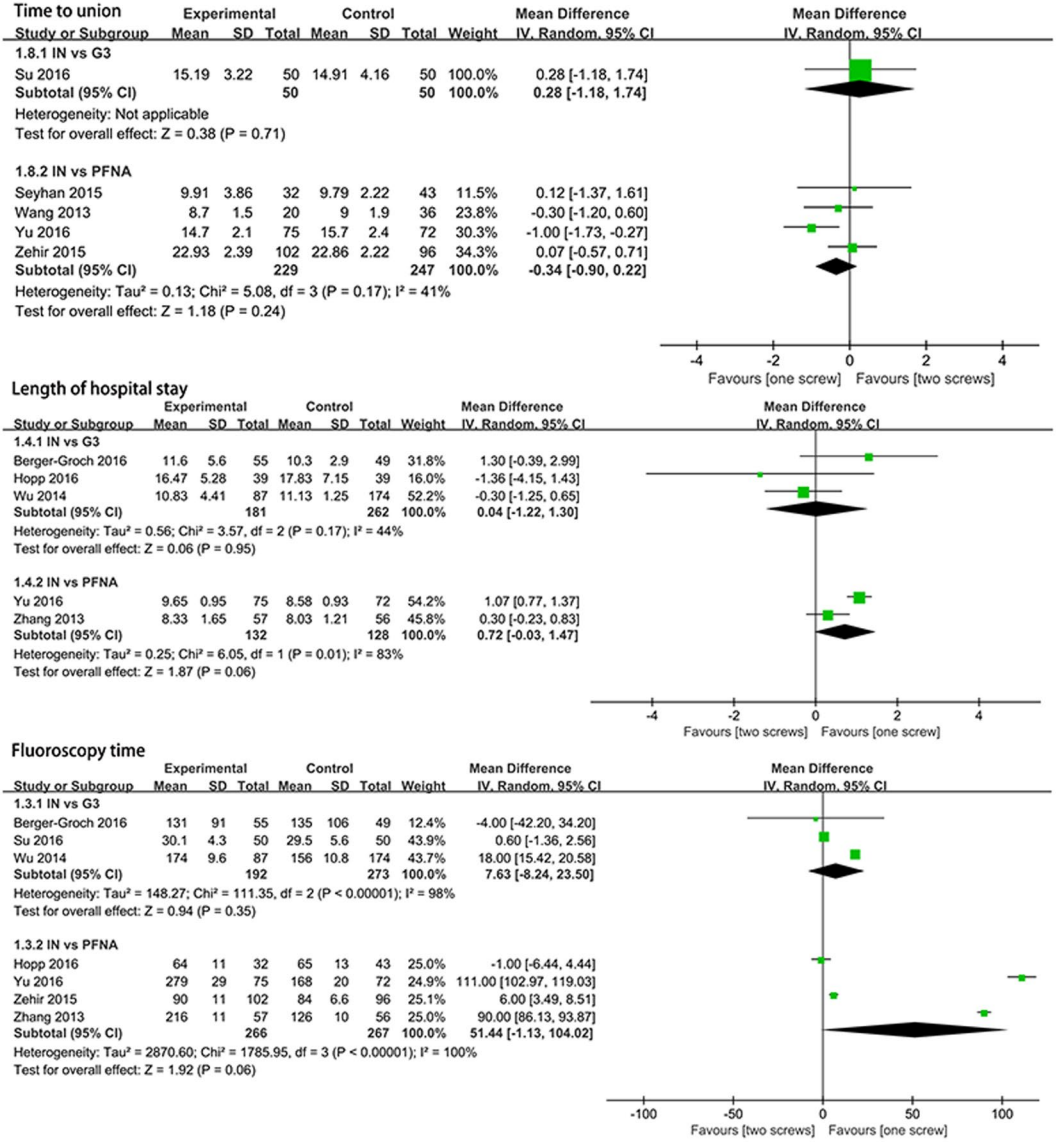
A forest plot diagram showed the time to union, length of hospital stay and fluoroscopy time.

Data from five studies with 576 patient reported the union time. Subgroup analysis showed that no significant differences were found in IT vs. Gamma 3 (MD = 0.28, 95%CI:[−1.18, 1.74], P = 0.71; Fig. 5) and IT vs. PFNA (MD = −0.34, 95%CI:[−0.9, 0.22], *P* = 0.24; Fig. 5) subgroup. Random effect model was used due to moderate heterogeneity in union time.

#### Length of hospital stay

Five articles with 703 patients showed the outcome of length of hospital stay. Subgroup analysis indicated that no significant differences were found in IT vs. Gamma 3 (MD = 0.04, 95%CI:[−1.22, 1.3], *P* = 0.95; Fig. 5) and IT vs. PFNA (MD = 0.72, 95%CI:[−0.03, 1.47], *P* = 0.06; Fig. 5) subgroup. We used random effect model due to the statistical heterogeneity.

#### Fluoroscopy time

A meta-analysis examined seven studies (998 patients) to assess fluoroscopy time. There were no significant differences in IT vs. Gamma 3 (MD = 7.63, 95%CI:[−8.24 23.5], *P* = 0.35; Fig. 5) and IT vs. PFNA (MD = 51.44, 95%CI:[−1.13, 104.02], *P* = 0.06; Fig. 5) subgroup. Due to the statistical heterogeneity, we used random-effect model to explain the pooled results.

#### Blood loss

**Figure 6:**
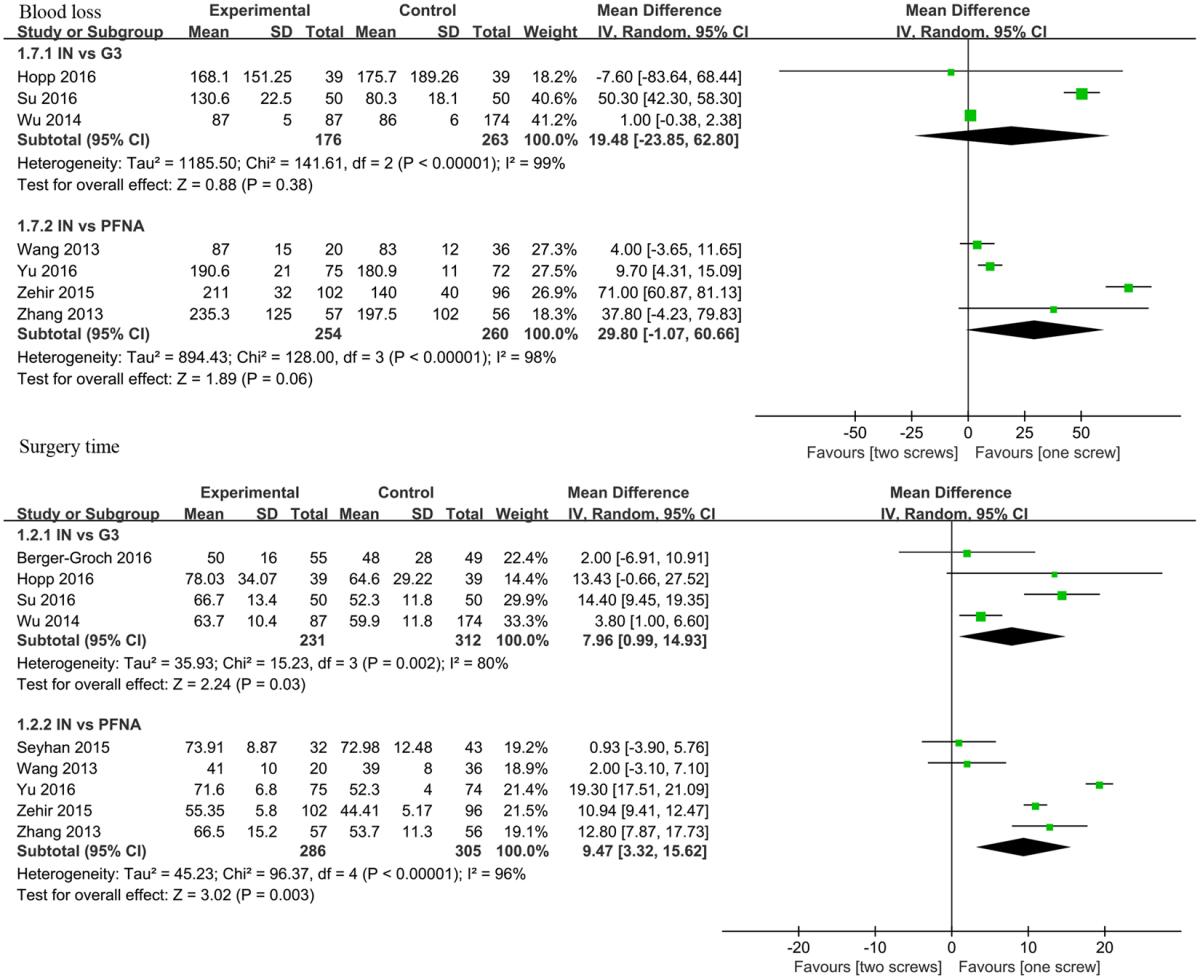
A forest plot diagram showed the blood loss, total complications and surgery time.

Subgroup analysis reported that no significant differences were found in IT vs. Gamma 3 (MD = 19.48, 95%CI:[−23.85, 62.8], *P* = 0.38; Fig. 6) and IT vs. PFNA (MD = 29.8, 95%CI:[−1.07, 60.66], *P* = 0.06; Fig. 6) subgroup. Due to statistical heterogeneity, we used random-effects model to explain the pooled results.

#### Surgery time

We extracted the data of surgery time from nine articles. Significant differences were found in IT vs. Gamma 3 (MD = 7.96, 95%CI:[0.99 14.93], *P* = 0.03; Fig. 6) and IT vs. PFNA (MD = 9.47, 95%CI:[3.32, 15.62], *P* = 0.0003; Fig. 6) subgroup. There was statistical heterogeneity between included studies, and we used random-effects model to explore the heterogeneity.

#### Complications

**Figure 7:**
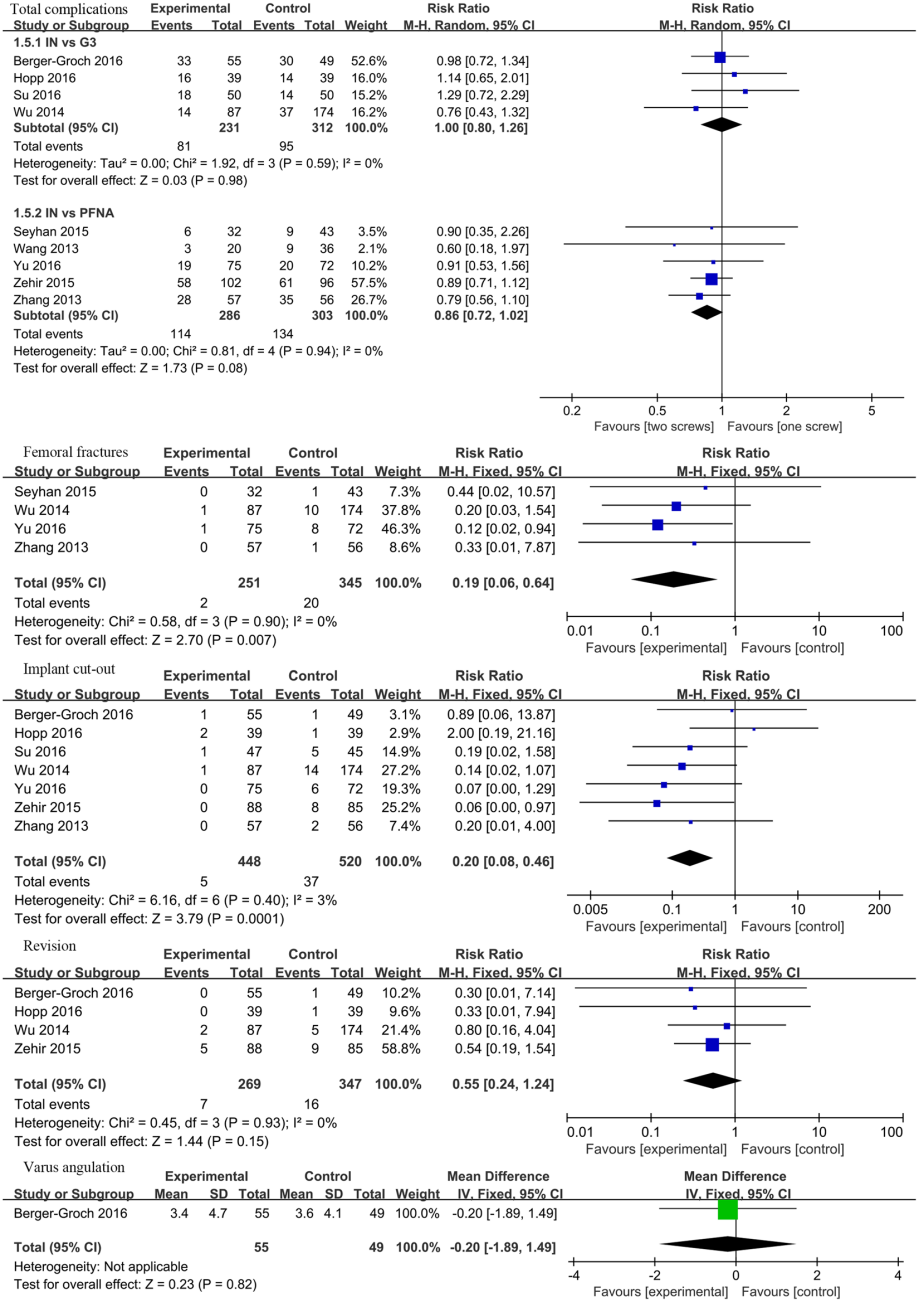
A forest plot diagram showed the local complications.

Complications were reported in all the included nine studies. Subgroup analysis indicated that no significant differences were found in IT vs. Gamma 3 (RR = 1, 95%CI:[0.8, 1.26], *P* = 0.98; Fig. 7) and IT vs. PFNA (RR = 0.86, 95%CI: [0.72, 1.02], *P* = 0.08; Fig. 7) subgroup.

Local complications such as implant cut-out, femoral fractures, revision surgery and varus angulation was also extracted from the included studies. Significant differences were found between IT and Gamma 3 or PFNA groups in implant cut-out (RR = 0.2, 95%CI: [0.08, 0.46], *P* = 0.001; Fig. 7) and femoral fractures (RR = 0.19, 95%CI: [0.06, 0.64], *P* = 0.007; Fig. 7). However, we failed to find any significant in revision surgery (RR = 0.55, 95%CI: [0.24, 1.24], *P* = 0.15; Fig. 7) and varus angulation (RR = −0.2, 95%CI: [−1.89, 1.49], *P* = 0.0.82; Fig. 7) between two groups. No statistical heterogeneity was found between included studies, and we used fixed-effect model to explore heterogeneity.

### Sensitivity analysis and publication bias

Sensitivity analysis (Supplemental Fig. 1) showed that no heterogeneity was found among included studies. The funnel plot was symmetric (Supplemental Fig. 2) and Egger’s test was conducted in our meta-analysis, and we found no significant difference in Egger’s test (*P* = 0.155).

## Discussion

There are still controversies about the optimal implant system to stabilize unstable intertrochanteric fractures, especially in the elderly patients with osteoporosis. Intramedullary nails system are one of the most commonly treated unstable intertrochanteric fractures in older adults, and the prevalence of this implant system will continue to rise as the population ages^23,24^. The IT nail system, introduced in 2005, has been reported to withstand higher loads in previous biomechanical study compared with 1-screw nailing system^25^. A previous clinical study reported that IT showed good clinical outcome and low complication rate in Asian patients^26^. However, when compared with 1-screw nailing system, patients treated with the IN nail showed similar HHS and experienced longer fluoroscopy and operative time^19^. It was not clear whether IT can provide better clinical outcomes. Therefore, there was a need for an evidence base or recommendations to help surgeons make clinical decisions.

The present meta-analysis was conducted to explore whether 2-screw intramedullary nail provided better clinical outcomes as did 1-screw nailing system used in intertrochanteric fractures. Our pooled data showed that 2-screw intramedullary nail was as effective to 1-screw nailing system in terms of HHS, union time, length of hospital stay, fluoroscopy time, blood loss and complications. However, less surgery time was used in 1-screw nailing system.

Functional assessment is a very important part of rehabilitation therapy. HHS was usually used to evaluate the results of hip surgery. HHS was the primary outcome assessed in our systematic review and meta-analysis. HHS score can comprehensively assess the function of patient after hip surgery. The pooled data showed that IT as effective for postoperative functional recovery and pain relieve in patients with intertrochanteric fractures as 1-screw nailing system. Recently, a cohort study have demonstrated that there were significant differences between the two groups for complications such as femoral neck shortening, local complications, fracture healing time and blood loss for unstable intertrochanteric fractures^20^. Seyhan *et al*. reported that the local complications in the PFNA group including reverse displacement rates of proximal screw, proximal femur shortening, and decrease in the varus angle were significantly higher than the InterTan group^9^. Although these studies showed positive results of IT, poor study design and short period of follow-up decreased the degree of evidence. Meanwhile, moderate heterogeneity and risk of bias should be considered when interpreting these findings. An RCT with five-years of follow up demonstrated that no statistically significant differences were found in functional outcomes between IT and 1-screw nailing groups^10^. This was consistent with our findings. In our meta-analysis, we failed to find any significant differences between the two groups in HHS between two groups. A prospective cohort study demonstrated that there were no statistically significant differences in complications, walking ability, Harris Hip Scores, and hip range of motion at one-year of follow-up between IT and PFNA group^22^. Therefore, compared with the 2-screw intramedullary nail group, 1-screw nailing system provided similar functional recovery for patients with intertrochanteric fractures. In addition, compliance of HHS for elderly population specially for patients with dementia should be considered.

Union time and complications are also important post-operative indicators to evaluate the functional recovery. A prospective cohort study have clearly shown that the IT is comparable to the Gamma 3 nail system in terms of HHS, time to union and implant-related complication rate for unstable intertrochanteric fractures^16^. Zehir *et al*.^21^ reported that there were no significant differences between two groups in fracture union time (IT vs. PFNA: 3.35 ± 2.01 vs. 3.29 ± 1.89 weeks, *p* = 0.43). The results of our meta-analysis are in consensus with the results of the findings mentioned above. The pooled data in our meta-analysis reported no significant difference in both the two-screw and 1–screw nail groups regarding union time, total postoperative complications, revision surgery and varus angulation. However, IT group showed less implant cut-out and femoral fractures than 1-screw group. What’s more, the quality of evidence was moderate. Therefore, we could confidently draw conclusions about these results.

We also pooled the data of perioperative outcomes such as blood loss, surgery time and fluoroscopy time. These outcomes are important for surgeons to assess the postoperative recovery of patients. Wu *et al*.^19^ reported that the surgery time and fluoroscopy time were significantly longer in IT group than Gamma3 group, and no statistical differences were observed in blood loss between two groups. Hopp *et al*.^16^ demonstrated that the mean operating time in the Gamma 3 group was 14 minutes shorter than in the IT group (64 vs. 78 min; *p* = 0.044), and the intraoperative blood loss (average 171.9 ml) were not significantly influenced. Taking these findings together, operating time in IT group was longer than the 1-screw nailing system, but the blood loss in two groups was similar. Length of hospital stay was also an important indicator to evaluate the postoperative functional recovery. No statistical significance was found in the length of hospital stay between the two groups in our meta-analysis. A prospective randomized study reported that there were no significant difference in mean hospital stay between IT (8.33 ± 1.65 days) and PFNA group (8.03 ± 1.21 days)^22^. Also, costs of InterTan should be considered. Prices will vary around the world and local costs of implants may influence choices of implants.

Also, there are some limitations in our systematic review and meta-analysis. Firstly, only nine studies of high quality with 1119 patients are included to perform meta-analysis; the sample size is relative small and if more RCTs are included, the test power for statistical analysis would be more convincing. Secondly, the follow-up of patient is different between included articles. One study conducted by Wang *et al*. reported the mean time of follow up is 4.6 months. Thirdly, heterogeneity between the included literatures may affect the results of our meta-analysis. A variety of factors including racial differences, surgery procedures, age differences, study design, anaesthesia factors and different time of follow-up may cause the heterogeneity. These factors between different studies are inevitable. Although some limitations exist in our study, relevant articles have been stringently screened according to the inclusion and exclusion criteria and the extracted data are of high quality.

## Conclusions

IT is not found to be superior to 1-scew nailing system in terms of HHS, blood loss, fluoroscopy time, union time, total postoperative complications and length of hospital stay. In conclusion, compared with IT, 1-scew nailing system shows similar functional recovery in unstable intertrochanteric fractures. Although, IT shows less implant cut-out and femoral fractures. However, since IT shows similar functional recovery, revision rate and longer surgery time, we conclude that it is not worthy of being recommended as an alternative intramedullary nail in intertrochanteric fractures.

## Electronic supplementary material

Supplementary Information
