# Supplementary material for: Comparison of clinical outcomes with InterTan vs Gamma nail or PFNA in the treatment of intertrochanteric fractures: A meta-analysis
Source: Sci Rep. 2017 Nov 21;7:15962. doi: 10.1038/s41598-017-16315-3 (PMC5698321; doi:10.1038/s41598-017-16315-3)

Comparison of clinical outcomes with InterTan vs Gamma nail or PFNA in the treatment of intertrochanteric fractures: A meta-analysis

Jian-xiong Ma [a, *], Ming-jie Kuang[a,b, *], Zheng-rui Fan [a,b, *], Fei Xing[a,b], Yun-long Zhao[a,b], Lu-kai Zhang[a], Heng-ting Chen[a,b], Chao Han[a,†], Xin-long Ma[a,b,†]

*These authors contributed equally to this work.

†Correspondence to Xin-long Ma and Chao Han.

[a] Biomechanics Labs of Orthopaedics Institute, Tianjin Hospital, Tianjin 300050, People's Republic of China

[b] Department of Orthopedics, Tianjin Medical University General Hospital, Tianjin 300052, People's Republic of China

Corresponding author: Xin-long Ma, Tianjin Hospital, Tianjin 300211, China, e-mail：2009021106@tmu.edu.cn, phone number: +86-13602179865.

Chao Han, Tianjin Hospital, Tianjin, 300050, China, e-mail: craborth@163.com, phone number: +86-13512862268

Name	Email
Jian-xiong Ma	mjx969@163.com
Ming-jie Kuang	seagullkmj@126.com
Zheng-rui Fan	517494450@qq.com
Fei Xing	lisomox@outlook.com
Yun-long Zhao	297286061@qq.com

Supplemental Table 1: Search strategy on PubMed

#1 Intertrochanteric Fractures

#2 Trochanteric Fractures

#3 "Hip Fractures"[Mesh]

#4 Search (#1 OR #2) OR #3

#5 Proximal femoral nail antirotation

#6 Gamma nail

#7 Intertan

#8 "Bone Nails"[Mesh]

#9 Search (#5 OR #6) OR #7

#10 random*

#11 Cohort study

#12 "Randomized Controlled Trial" [Publication Type]

#13 "Randomized Controlled Trials as Topic"[Mesh]

#14 "Cohort Studies"[Mesh]

#15 Search (#10 OR #11 OR #12 OR #13 OR #14 OR #15)

#16 Search (#4 AND #9 AND # 15)

Supplemental Table 2. Study quality assessment using Newcastle-Ottawa scale for cohort studies.

Studies/year	Selection: representativeness of the exposed cohort	selection of the non-exposed cohort	ascertainment of exposure	outcome of interest was not present at start of study	comparability of cohorts on the basis of the design or analysis	Outcome: assessment of outcome	was follow-up long enough for outcomes to occur?	adequacy of follow up of cohorts	Total score
Wang 2011	*	*	–	*	** (age, gender, BMI etc)	*	*	*	8
Wu 2014	*	*	*	*	* (age, gender, etc)	*	*	*	9
Yu 2016	*	*	–	*	* (age, gender, etc)	*	*	–	6
Zehir 2015	*	*	*	*	* (age,etc)	*	*	*	8

Supplemental table 3: GRADE of the evidence

Quality assessment							No of patients		Quality	Importance
No of studies	Study design	Risk of bias	Inconsistency	Indirectness	Imprecision	Other consideration	Outcome	Control		
**HHS: two-screw vs. 1-screw nailing system**										
9	randomised trials and cohort studies	serious	serious	no serious indirectness	no serious imprecision	none	517	612	⊕⊕OO LOW	CRITICAL
**Surgery time (min): two-screw vs. 1-screw nailing system**										
9	randomised trials and cohort studies	serious	serious	no serious indirectness	no serious imprecision	none	517	612	⊕⊕OO LOW	IMPORTANT
**fluoroscopy time(s): two-screw vs. 1-screw nailing system**										
7	randomised trials and cohort studies	very serious	very serious	no serious indirectness	no serious imprecision	none	458	540	⊕OOO VERY LOW	IMPORTANT
**length of hospital stay: two-screw vs. 1-screw nailing system**										
3	randomised trials and cohort studies	serious	serious	no serious indirectness	no serious imprecision	none	313	390	⊕⊕OO LOW	IMPORTANT
**Complications: two-screw vs. 1-screw nailing system**										
9	randomised trials and cohort studies	serious	no serious	no serious indirectness	no serious imprecision	none	195/517	229/612	⊕⊕⊕O	IMPORTANT

Supplemental Fig 1

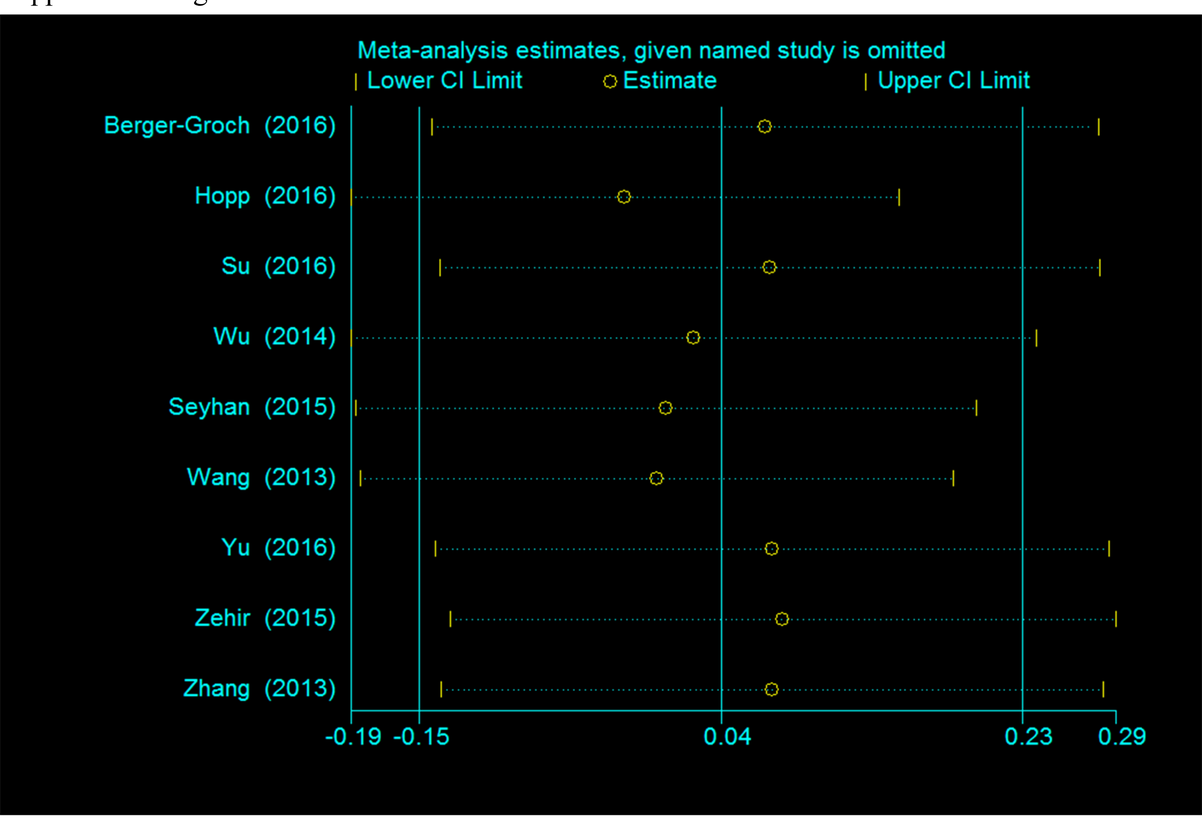

Supplemental Fig 2

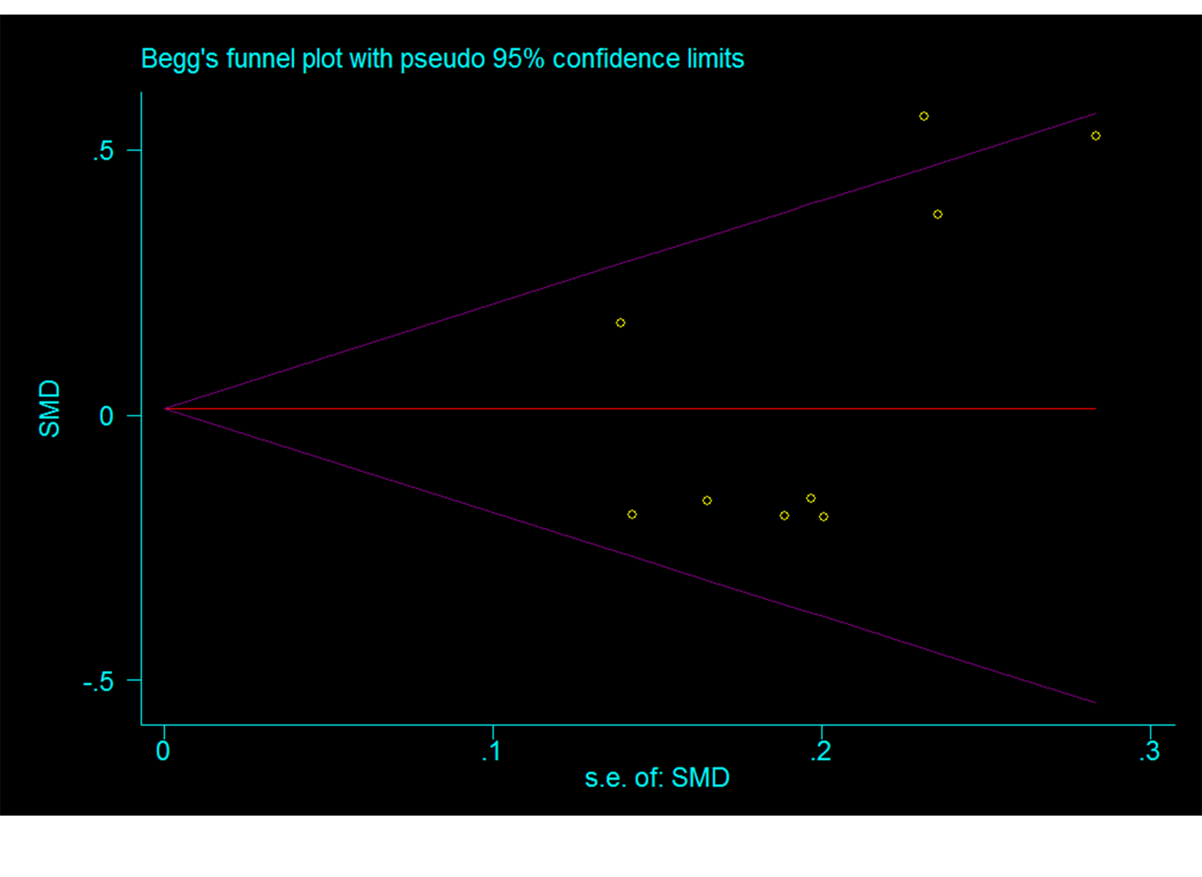

Supplement: Supplementary file 1 — Supplementary Information [file 41598_2017_16315_MOESM1_ESM.pdf]
